# Sub-Cutaneous Morphia

**Published:** 1874-01

**Authors:** 


Elsewhere, in "*Our Exchanges*," we reprint an ingenious suggestion for the administration of subcutaneous morphia, which we doubt not will strike the attention of many of our readers favorably, as it does our own, on account of its great simplicity and apparent utility. We beg leave to suggest to all such a few thoughts, viz:

1. Let the thread be *slightly moistened* for such a length as may be designed for lodgment beneath the skin; place one-eighth grain acetate morphia upon glazed paper or glass, and roll the moistened portion of thread in the morphia until it is *thoroughly* absorbed.

2. Let the thread be white sewing silk, which is loosely twisted, not the hard twisted and glazed Coates' cotton.

3. Let the needle be of such size as to carry the silk *easily*, that the solution may not be stripped out by the contraction of the cutaneous orifice.

4. Let the needles be long and tapering, such as are technically called "Sharp's" by the manufacturers.

5. Let the needle be oiled, and then *lightly wiped* before using.

6. When a larger dose is needed, use two or more threads in separate punctures.

7. The threads may be retained longer, if necessary to secure absorption.

We shall be happy to receive the experience of our readers in the future use of this method.

